# EcoDetect-YOLOv2: A High-Performance Model for Multi-Scale Waste Detection in Complex Surveillance Environments

**DOI:** 10.3390/s25113451

**Published:** 2025-05-30

**Authors:** Jing Su, Ruihan Chen, Mingzhi Li, Shenlin Liu, Guobao Xu, Zanhong Zheng

**Affiliations:** 1 School of Mathematics and Computer, Guangdong Ocean University, Zhanjiang 524088, China; 2 School of Microelectronics and Communication Engineering, Chongqing University, Chongqing 400044, China

**Keywords:** multi-scale waste detection, YOLOv8s, ResGhostCSP, P2, EMA, Dysample

## Abstract

Conventional waste monitoring relies heavily on manual inspection, while most detection models are trained on close-range, simplified datasets, limiting their applicability for real-world surveillance. Even with surveillance imagery, challenges such as cluttered backgrounds, scale variation, and small object sizes often lead to missed detections and reduced robustness. To address these challenges, this study introduces EcoDetect-YOLOv2, a lightweight and high-efficiency object detection model developed using the Intricate Environment Waste Exposure Detection (IEWED) dataset. Building upon the YOLOv8s architecture, EcoDetect-YOLOv2 incorporates a small object detection P2 detection layer to enhance sensitivity to small objects. The integration of an efficient multi-scale attention (EMA) mechanism prior to the P2 head further improves the model’s capacity to detect small-scale targets, while bolstering robustness against cluttered backgrounds and environmental noise, as well as generalizability across scale variations. In the feature fusion stage, a Dynamic Upsampling Module (Dysample) replaces traditional nearest-neighbor upsampling to yield higher-quality feature maps, thereby facilitating improved discrimination of overlapping and degraded waste particles. To reduce computational overhead and inference latency without sacrificing detection accuracy, Ghost Convolution (GhostConv) replaces conventional convolution layers within the neck. Based on this, a GhostResBottleneck structure is proposed, along with a novel ResGhostCSP module—designed via a one-shot aggregation strategy—to replace the original C2f module. Experiments conducted on the IEWED dataset, which features multi-object, multi-class, and highly complex real-world scenes, demonstrate that EcoDetect-YOLOv2 outperforms the baseline YOLOv8s by 1.0%, 4.6%, 4.8%, and 3.1% in precision, recall, mAP_50_, and mAP_50:95_, respectively, while reducing the parameter count by 19.3%. These results highlight the model’s effectiveness in real-time, multi-object waste detection, providing a scalable and efficient tool for automated urban and digital governance.

## 1. Introduction

With the rapid advancement of the global economy and accelerating urbanization, municipal solid waste (MSW) generation has continued to rise, posing a significant challenge to ecological sustainability and public health. Projections indicate that annual global waste production will surge from 2.01 billion metric tons in 2016 to 3.4 billion metric tons by 2050 [1], exerting severe environmental and societal pressures. Efficient and scientifically informed waste management is critical for mitigating environmental pollution and reducing the risk of disease transmission [2], while also constituting a fundamental component of sustainable development strategies. In urban environments and public spaces, MSW primarily comprises paper, plastics, and metals [3], among which plastic waste, due to its widespread distribution, persistence, and potential ecological toxicity, presents a particularly severe threat to both the environment and human health [4].

Extensive research has demonstrated that, in the absence of effective waste management, numerous environmental and societal issues may arise, including urban aesthetic degradation, proliferation of pathogenic microorganisms, air and water pollution, and even long-term adverse impacts on tourism and urban economies [5]. Despite increasing public awareness of the hazards associated with waste pollution, efficient MSW management remains a formidable challenge worldwide, particularly in urban areas of developing nations and certain developed countries [6]. The extensive and dynamic distribution of waste renders traditional manual inspection-based collection and cleaning methods costly, susceptible to omissions, and potentially hazardous to sanitation workers’ occupational health. Therefore, the development of a lightweight and highly generalizable algorithm, capable of real-time waste detection and integration with urban surveillance systems, is of paramount importance for reducing manual inspection costs, advancing automation in MSW management, and fostering digital economic growth.

In recent years, the emergence of deep convolutional neural networks has revolutionized object detection by enabling end-to-end learning of discriminative feature representations directly from raw image data. Through hierarchical extraction of semantic information ranging from low- to high-level cues, contemporary detectors have surpassed the limitations of hand-crafted features, delivering substantial improvements in both accuracy and computational efficiency [7,8,9,10,11]. Among these approaches, one-stage architectures—such as the Single Shot MultiBox Detector (SSD) [12] and the You Only Look Once (YOLO) series [13]—have proven particularly effective for real-time applications, simultaneously predicting object classes and bounding boxes in a single forward pass.

Building upon these advancements, a growing body of research has focused on adapting one-stage detectors to the unique challenges posed by waste detection tasks. Liu et al. [14] proposed a YOLOv5-based model capable of effective detection in simple backgrounds (e.g., lawns, pavements). Patel et al. [15] developed various waste detection models; however, their effectiveness remained constrained due to the limited scale of available datasets. Mao et al. [16] designed a single-object waste detector based on YOLOv3 using a dataset of recyclable waste from Taiwan. Li et al. [17] introduced a cascaded detection framework combining SSD, YOLOv4, and Faster R-CNN to enhance detection reliability and reduce false positives.

Despite these advances, existing waste detection models are predominantly trained on relatively simple scenarios, facing significant challenges when deployed in surveillance camera perspectives. These challenges include the difficulty of detecting small objects, interference from complex backgrounds, occlusion of waste items, and high variability in object morphology, all of which contribute to substantial limitations in detection accuracy and real-time performance. Furthermore, many state-of-the-art detection models entail high computational complexity, making them unsuitable for real-time monitoring applications.

To address these challenges, this study introduces EcoDetect-YOLOv2, an optimized waste detection algorithm based on an improved YOLOv8 architecture, leveraging the waste detection dataset constructed by Liu et al. [18] in a surveillance camera environment. The primary contributions of this study are as follows:

(1) A novel Cross-Stage Partial (CSP) module, ResGhostCSP, was designed by integrating the GhostConv operation with a One-Shot Aggregation strategy. This approach reduces computational complexity and inference time while maintaining detection accuracy.
(2) A new small-object detection layer, P2, was incorporated to retain finer-scale details and positional information, thereby improving the accuracy and efficiency of small-object waste detection.
(3) An EMA mechanism was introduced before the P2 detection head, enhancing model robustness in complex and noisy environments while improving cross-scale object generalization.
(4) In the neck network, traditional nearest-neighbor upsampling was replaced with Dysample upsampling, generating higher-quality feature maps and improving the model’s ability to distinguish overlapping waste objects.

The remainder of this paper is structured as follows: Section 2 provides an overview of the original YOLOv8s architecture, multi-scale feature fusion techniques, and attention mechanisms. Section 3 describes the dataset and the proposed EcoDetect-YOLOv2 algorithm. Experimental procedures and analysis are detailed in Section 4. Finally, conclusions are drawn in Section 5.

## 2. Related Work

### 2.1. YOLOv8

As one of the most advanced object detection algorithms to date, YOLOv8 demonstrates exceptional performance in both detection accuracy and computational efficiency, surpassing the majority of existing object detection methods. Therefore, this study selects YOLOv8 as the baseline model for comparative analysis.

**Figure 1:**
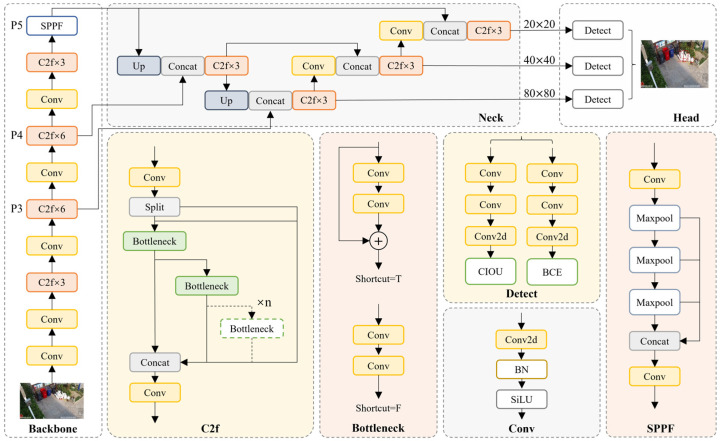
Overall architecture of YOLOv8.

As illustrated in Figure 1, YOLOv8 employs Darknet53 as its backbone network and integrates the Path Aggregation Feature Pyramid Network (PAFPN) during the feature fusion stage to enhance multi-scale feature representation. In the detection head design, YOLOv8 adopts an anchor-free mechanism, eliminating the need for predefined anchor boxes. This significantly reduces the number of bounding box predictions, thereby improving the execution speed of Non-Maximum Suppression (NMS). As NMS is computationally intensive in the post-processing stage for object selection, the anchor-free design effectively decreases the computational burden during inference, enhancing real-time detection performance.

Regarding data augmentation, YOLOv8 employs Mosaic augmentation, a technique that enriches training data by randomly cropping and stitching multiple images together, thereby improving the model’s generalization capability across diverse scenarios and objects. However, Mosaic augmentation may lead to overfitting during training. To mitigate this issue, this study disables Mosaic augmentation during the final 10 epochs of training, allowing the model to converge on the original dataset without augmented distortions, thereby alleviating potential drawbacks associated with Mosaic augmentation.

For loss computation, YOLOv8 incorporates the TaskAlignedAssigner from Task-Aligned Object Detection (TOOD) [19] as its dynamic target assignment strategy, optimizing the target matching mechanism. The target assignment in TOOD is formulated as follows:

(1) $$t=s^{\alpha }\times u^{\beta }$$

where s denotes the predicted score corresponding to the ground truth class, u represents the Intersection over Union (IoU) between the predicted and ground truth bounding boxes, t is the resulting matching quality metric that jointly reflects classification confidence and localization accuracy; the exponent α governs the relative weighting of the classification score, while β modulates the influence of the IoU-based localization term. The loss function is defined as:

(2) $$loss\left(o,t\right)=-\frac{1}{n}\left(\sum_{i}\left(t\left[i\right]*\log\left(o\left[i\right]\right)+\left(1-t\left[i\right]\right)*\log\left(1-o\left[i\right]\right)\right)\right)$$

where n denotes the total number of samples used in the loss computation, i indexes each sample, $o\left[i\right]$ represents the predicted probability produced by the model, and $t\left[i\right]$ is the ground truth label for the i-th sample, and the ground truth probability is implicitly defined by the label. This task-aligned strategy enables dynamic adjustment of target assignment, thereby improving the precision of bounding box matching and enhancing the model’s overall detection performance.

Compared to the widely adopted YOLOv5, YOLOv8 refines several architectural components. The first convolutional layer kernel size transitions from 6 × 6 to 3 × 3, while the C3 module is replaced with the C2f module, which incorporates additional skip connections and feature splitting operations. Additionally, two convolutional layers are removed to simplify the neck module. A significant modification is observed in the head design, which transitions from a coupled head to a decoupled head, replacing the anchor-based approach of YOLOv5 with an anchor-free paradigm.

### 2.2. Attention Mechanism

The attention mechanism, inspired by human visual perception and neural information processing, has been extensively applied in deep learning [20]. This mechanism dynamically allocates computational resources, allowing models to focus on critical feature information while suppressing irrelevant signals, thereby improving feature representation and task awareness. Furthermore, attention mechanisms effectively reduce model redundancy, enhance robustness against noise, and improve network generalization.

In conventional neural networks, all input features are typically treated equally, leading to computational inefficiency and reduced generalization capability [21]. However, in many practical tasks, only a subset of input features significantly influences the final prediction. Treating all features uniformly can introduce unnecessary computational overhead. Additionally, deep neural networks are often regarded as “black-box” models with limited interpretability. The incorporation of attention mechanisms enables weight visualization, enhancing model interpretability and facilitating network optimization.

Attention mechanisms have demonstrated outstanding performance across various deep learning tasks. For instance, in image classification, attention mechanisms enable models to focus on discriminative regions, improving classification accuracy. In text summarization, these mechanisms help identify key information in source text, generating more precise and concise summaries [22]. By optimizing feature selection and information filtering, attention mechanisms have achieved significant advancements in computer vision, natural language processing, and speech recognition, establishing themselves as a pivotal research direction in deep learning.

### 2.3. Lightweight Networks

Lightweight neural networks are designed to maintain high task performance while reducing parameter size and computational complexity, making them suitable for resource-constrained environments. These networks leverage various optimization strategies, such as structural simplification, parameter redundancy elimination, and computation graph optimization, ensuring efficient deployment on mobile devices, embedded systems, and edge computing platforms. By enhancing inference speed and reducing storage overhead, lightweight networks strike a balance between accuracy and real-time performance, making them highly applicable in fields such as intelligent surveillance, autonomous driving, and industrial inspection.

To minimize computational cost while maintaining detection efficiency, several lightweight strategies have been proposed. One approach focuses on reducing the precision of network weights to decrease storage requirements and enhance computational efficiency [23]. Another method involves pruning redundant parameters to optimize computation flow. For example, MADNet [24], a compact lightweight network, employs dense connections to enhance multi-scale feature representation and feature correlation learning. Additionally, Liu et al. [25] introduced a network architecture integrating dilated convolutions and attention mechanisms, leveraging multi-scale pooling operations to improve semantic information encoding. However, these methods primarily focus on compressing pre-trained models or training small-scale networks, which may impact overall performance equilibrium.

Addressing these challenges, this study proposes an optimized lightweight detection model, EcoDetect-YOLOv2, by extending the GhostConv structure with GhostResBottleneck and ResGhostCSP modules. This approach significantly reduces computational complexity and inference time while preserving detection accuracy, thereby achieving a superior lightweight object detection framework.

## 3. Proposed Methodology

### 3.1. EcoDetect-YOLOv2 Object Detection Model

**Figure 2:**
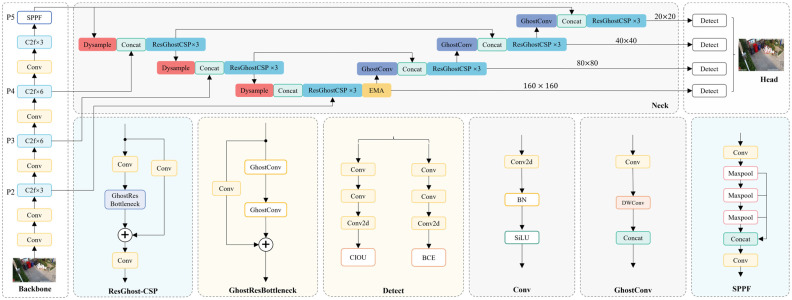
Overall Network Architecture of EcoDetect-YOLOv2.

In the context of waste detection through surveillance cameras, YOLOv8 encounters limitations in detection accuracy and its capacity for detecting small objects. To address these challenges, this study selects YOLOv8s as the baseline model and proposes an efficient, lightweight object detection network—EcoDetect-YOLOv2. The overall architecture of EcoDetect-YOLOv2 is depicted in Figure 2.

Specifically, to retain finer details and positional information, an additional small object detection layer, P2, is incorporated, enhancing both the detection efficiency and accuracy for small-sized waste. Within the Neck structure, an efficient multi-scale attention (EMA) mechanism is introduced before the P2 detection head, significantly improving the capability for detecting small objects, robustness to complex backgrounds and noise, and generalization across varying object scales. Furthermore, lightweight Dysample upsampling replaces the conventional nearest-neighbor upsampling, generating higher-quality feature maps and improving the model’s ability to distinguish overlapping and worn particles during feature fusion.

Additionally, this study introduces the GhostResBottleneck structure based on GhostConv and designs an efficient cross-stage partial (CSP) module, ResGhostCSP, using a one-shot aggregation strategy. These enhancements ensure high detection accuracy while significantly improving computational efficiency, facilitating lightweight feature extraction and fusion.

### 3.2. Enhancements in P2 Small Object Detection Layer

The original backbone structure of YOLOv8 extracts features through a top-down hierarchical downsampling process. As network depth increases, high-level semantic feature representations are enhanced, yet feature information for smaller objects is significantly reduced. Consequently, YOLOv8 struggles to learn distinctive features for small objects in the Neck layer, leading to potential missed detections in the three detection heads of the Head layer.

The standard YOLOv8 network comprises three detection heads. When processing an input image of size 640 × 640, the multi-stage downsampling in the Backbone and subsequent feature fusion in the Neck produce detection feature maps of 20 × 20, 40 × 40, and 80 × 80, which correspond to detecting objects larger than 32 × 32, 16 × 16, and 8 × 8 pixels, respectively. However, objects smaller than 8 × 8 pixels remain challenging to detect, resulting in missed detections.

From the perspective of surveillance cameras, waste exposure detection frequently involves numerous small objects, particularly paper debris, which occupy only a few pixels within an image. Relying solely on the P3 detection head risks missing a significant number of these small targets. To mitigate this issue, this study incorporates an additional P2 (160 × 160) detection head into the network, improving the model’s ability to recognize fine-grained objects.

### 3.3. Design of GhostResBottleneck and ResGhostCSP Based on GhostConv

#### 3.3.1. GhostConv

**Figure 3:**
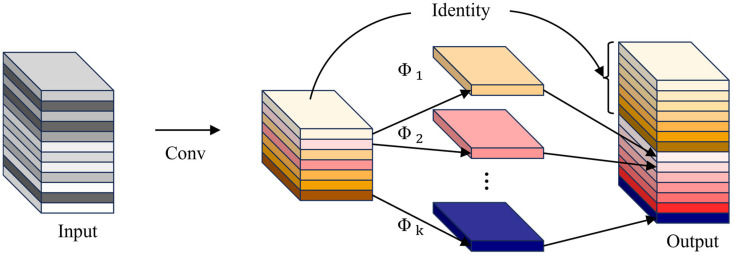
Structure of GhostConv.

Feature extraction in deep neural networks often generates a substantial number of redundant feature maps, leading to high computational costs. Although these redundant features are essential for data representation, they impose significant computational overhead. Inspired by GhostNet [26], which demonstrated the effectiveness of GhostConv in reducing computational costs, this study adopts the GhostConv mechanism to enhance computational efficiency during feature space expansion. GhostConv generates additional feature maps at a reduced computational expense, thereby lowering memory consumption during intermediate feature mapping. The fundamental structure of GhostConv is shown in Figure 3.

#### 3.3.2. GhostResBottleneck and ResGhostCSP Structure Design

**Figure 4:**
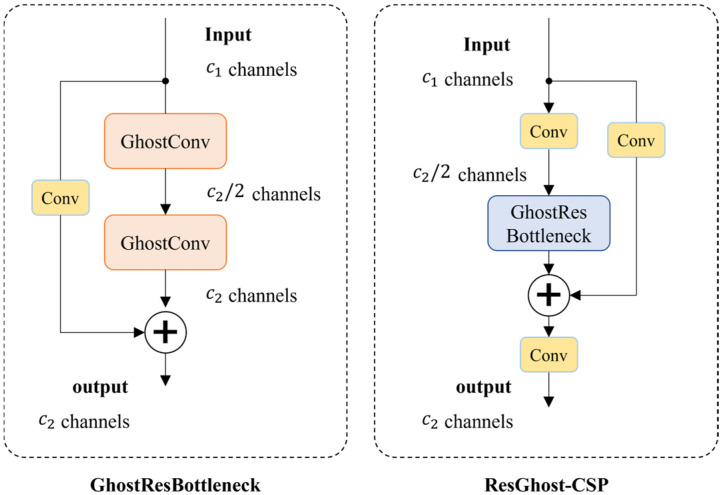
Structural Diagrams of GhostResBottleneck and ResGhostCSP.

GhostConv achieves approximately 50% of the computational cost of standard convolution (Conv) while maintaining comparable feature learning capability. Based on GhostConv, this study proposes the GhostResBottleneck structure, which integrates GhostConv with residual connections. The specific architecture of GhostResBottleneck is illustrated in Figure 4. Furthermore, to enhance the feature learning capability of CNNs, this study incorporates generalized deep learning optimization strategies [27,28,29] and employs a one-shot aggregation strategy to design an efficient, hardware-friendly cross-stage partial (CSP) module—ResGhostCSP. This module maintains high accuracy while effectively reducing computational complexity and inference time, as depicted in Figure 4.

To ensure effective feature extraction and improve network stability, residual connections are incorporated into both the GhostResBottleneck and ResGhostCSP structures. Residual connections mitigate training issues in deep networks, such as overfitting, gradient vanishing, and gradient explosion. The mathematical formulation is as follows:

(3) $$Output=F\left(Input,\left\{W_{i}\right\}\right)+Input,$$

where Input and Output denote the input and output of the residual block, respectively, while $F\left(Input,\left\{W_{i}\right\}\right)$ represents the nonlinear transformation applied to the input features, including convolution and activation functions. Compared to conventional feature concatenation (Concat) operations, residual connections alleviate gradient-related issues during deep network training, thereby enhancing model stability.

By combining the low-cost feature generation advantages of GhostConv with the efficient feature transmission capabilities of residual connections, GhostResBottleneck and ResGhostCSP reduce computational resource consumption while improving feature extraction. These advancements provide a robust foundation for designing lightweight, high-efficiency networks.

### 3.4. Efficient Multi-Scale Attention (EMA)

Efficient Multi-scale Attention (EMA), proposed by Ouyang et al. [30], is a multi-scale attention module designed for cross-spatial learning. Unlike conventional convolutional downsampling approaches, EMA selectively reshapes certain channels into batch channels for dimensionality reduction while employing cross-spatial learning strategies for multi-scale feature extraction. Convolutional operations are widely utilized in object detection and semantic segmentation due to their superior feature learning capabilities. However, as the depth of convolutional networks increases, memory consumption and computational complexity grow significantly. Furthermore, the translation-invariant nature of convolutions can lead to feature localization, limiting the effective modeling of global information. Introducing attention mechanisms within deep networks can enhance feature discrimination.

**Figure 5:**
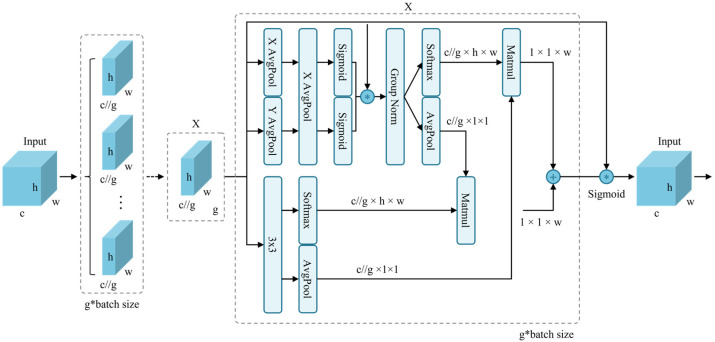
Structure of the EMA attention mechanism, where G represents the number of sub-features, X Avgpool indicates one-dimensional horizontal average pooling, and Y Avgpool denotes one-dimensional vertical average pooling.

To improve efficiency in waste detection from surveillance camera perspectives and to fully integrate multi-scale spatial and channel features, EMA is incorporated into the proposed model. Given an input feature map, the input tensor is defined as follows:

(4) $$X\in R^{C\times H\times W},$$

where C represents the number of channels, and H and W denote the spatial dimensions of the input feature map. The EMA mechanism (Figure 5) first partitions X into G groups of sub-features along the channel dimension:

(5) $$X=\left[\begin{array}{c} X_{0},X_{i},\ldots ,X_{G-1} \end{array}\right],X_{i}\in R^{C//G\times H\times W},$$

The core objective of EMA is to learn feature semantics across multiple scales. To achieve this, EMA employs a multi-scale feature extraction strategy utilizing two 1 × 1 branches and one 3 × 3 convolutional kernel in parallel, enhancing the fusion of multi-scale features. The 1 × 1 branches leverage global average pooling, while the 3 × 3 branch extracts features across multiple paths to capture inter-channel dependencies and reduce computational complexity. This mechanism encodes features and integrates them along the height dimension. Without reducing the number of channels, it applies shared 1 × 1 convolution and partitions the output into two vectors, employing a nonlinear sigmoid function to establish cross-channel interactions. Meanwhile, the 3 × 3 branch facilitates feature interactions through convolution operations, preserving spatial structural information. The spatial information of the three branches is then encoded using two-dimensional global average pooling:

(6) $$R_{1}^{1\times C//G}\times R_{3}^{C//G\times HW},$$

where the two-dimensional average pooling operation is computed as:

(7) $$Z_{c}=\frac{1}{H\times W}\sum_{j}^{H}\sum_{i}^{W}x_{c}\left(j,i\right).$$

To further enhance computational efficiency; EMA integrates softmax functions with average pooling operations for linear transformations; ultimately outputting a set of spatial attention weights to enhance feature representation. By effectively modeling multi-scale spatial and channel feature relationships; EMA ensures both accuracy and computational efficiency in waste recognition from surveillance perspectives. The core advantage of EMA lies in its efficient learning of feature semantics while integrating multi-scale information, offering an innovative solution for waste detection in complex environments

### 3.5. DySample Upsampling

DySample enhances computational resource efficiency by avoiding dynamic convolution operations and instead adopting a point-based sampling method for upsampling. In the YOLOv8 model, conventional upsampling methods typically require significant computational resources and parameters, limiting lightweight deployment in surveillance scenarios and thereby affecting small-object waste detection performance. In real-world monitoring applications, small waste objects, such as paper scraps, often occupy minimal pixel areas and are susceptible to pixel distortion, leading to the loss of fine details and posing challenges for feature learning. To address these issues, a lightweight and efficient dynamic upsampling method, DySample, is introduced as a substitute for conventional upsampling techniques. DySample improves small-object detection performance even under low-quality surveillance imaging conditions. The core principle combines point-based sampling strategies with learned sampling methods to achieve efficient upsampling, reducing computational burden while enhancing image resolution and overall model performance.

**Figure 6:**
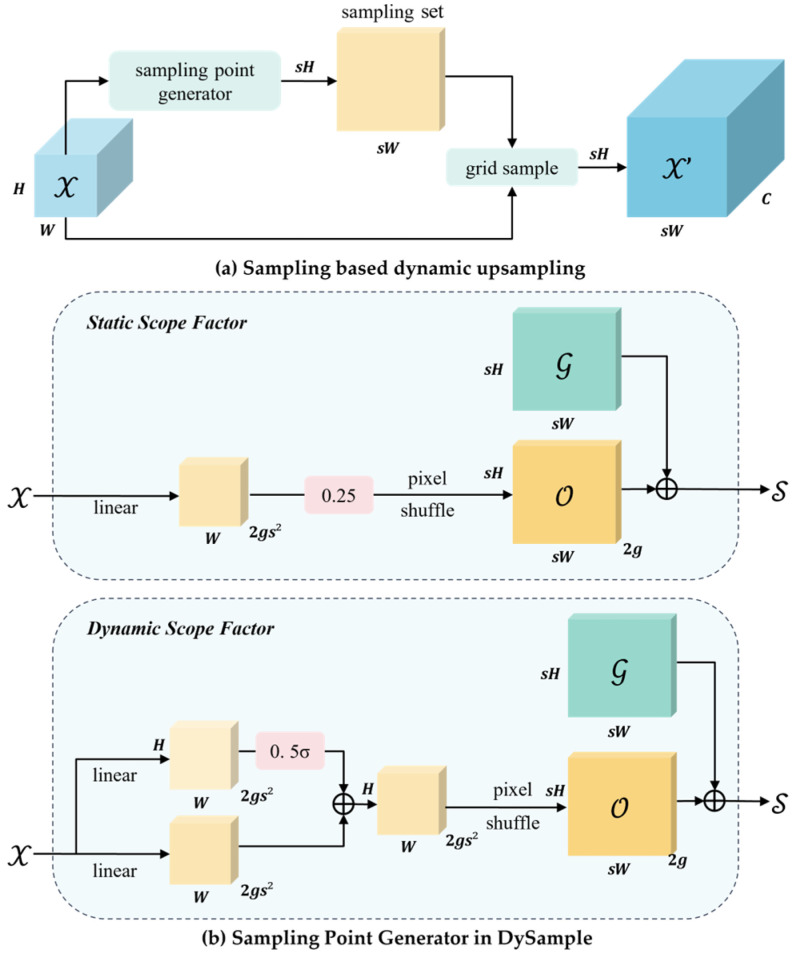
DySample module architecture demonstrating the point-based dynamic upsampling mechanism and design.

Figure 6 illustrates the point-based dynamic upsampling mechanism and modular design of DySample. The sampling point generator (Figure 6a) is a crucial component. Given an input feature map X of dimensions $C\times H_{1}\times W_{1}$ and a sampling set δ of size $2\times H_{2}\times W_{2}$, where the first two dimensions represent x and y coordinates, the grid_sample function resamples X using coordinates from δ. This process employs bilinear interpolation to generate a new feature map $X^{\prime }$ of size $C\times H_{2}\times W_{2}$:

(8) $$X^{\prime }=grid\_sample\;\left(X,\delta \right).$$

Assuming an upsampling factor of s, the input feature map has dimensions C×H×W. To implement upsampling, a linear transformation layer is first applied, taking an input channel size of and outputting $2s^{2}$ channels, resulting in an offset matrix O of size $2s^{2}\times H\times W$. The pixel shuffle algorithm then rearranges O into dimensions 2×sH×sW, generating the final sampling set δ:

(9) $$O=linear\left(X\right),$$

(10) δ=G+O.

While the description of the reshape operation is omitted, the final upsampled feature map $X^{\prime }$, derived from δ and the function grid_sample, has dimensions C×sH×sW as defined in Equation (9). Figure 6 further illustrates the DySample module’s dynamic upsampling mechanism and overall design.

## 4. Experimental Results and Discussion

### 4.1. Datasets

**Figure 7:**
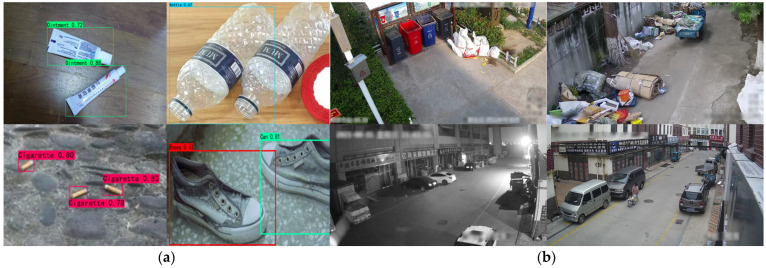
Example examples from (**a**) widely adopted waste-detection benchmarks and (**b**) the Intricate Environment Waste Exposure Detection dataset.

To evaluate the effectiveness of the proposed model, this study employs the Intricate Environment Waste Exposure Detection dataset introduced by Liu et al. [18] for performance assessment of EcoDetect-YOLOv2 and other benchmark models. This dataset encompasses nine categories of garbage detection targets, including paper trash, plastic trash, snakeskin bags, packed trash, stone waste, sand waste, cartons, foam trash, and metal waste. Representative samples from established waste detection benchmarks (Figure 7a) are juxtaposed with imagery from the proposed Intricate Environment Waste Exposure Detection (IEWED) dataset (Figure 7b). In contrast to conventional datasets—typically composed of close-range images with homogeneous backgrounds—IEWED comprises surveillance footage acquired from elevated, oblique viewpoints under variable lighting conditions and within dynamically evolving scenes. This real-world setting introduces three principal challenges for automated detection: (1) multi-scale object representation, as waste items often occupy a small, transient fraction of the visual field; (2) substantial background heterogeneity, characterized by occlusions and complex textures; and (3) inherently lower resolution and higher noise levels. These conditions necessitate the development of detection frameworks with enhanced sensitivity, accurate spatial localization capabilities, and robust feature extraction mechanisms resilient to scale variation and noise interference.

**Figure 8:**
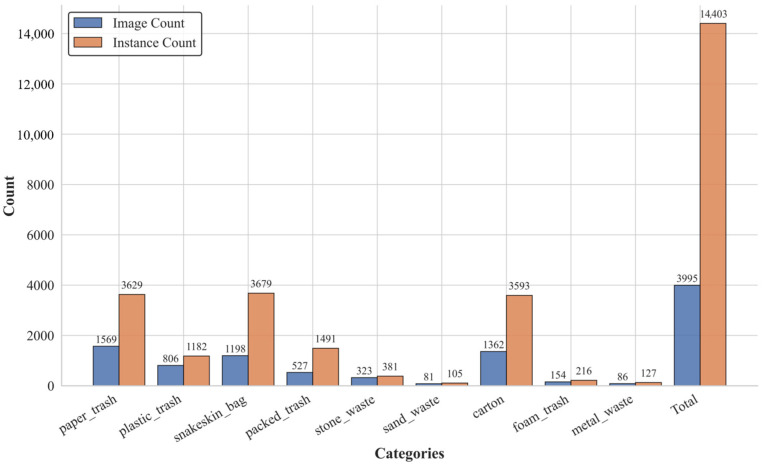
Category distribution of the dataset: the dark blue bars represent the number of images per category, while the dark orange bars indicate the instance count for each garbage category.

Figure 8 illustrates the composition of the dataset, providing a detailed account of both the number of images (“Image Count”) and the number of annotated object instances (“Instance Count”) across categories. To preserve the intrinsic class distribution, a hierarchically stratified sampling strategy was adopted. The dataset was partitioned into training and testing subsets following an 8:2 split, ensuring proportional representation of each category and its subordinate levels during both model training and evaluation.

### 4.2. Experimental Evaluation Metrics

#### 4.2.1. Precision and Recall

Precision (*P*) quantifies the proportion of correctly identified positive samples among all predicted positive instances, computed as follows:

(11) $$Precision=\frac{TP}{FP+FP}.$$

Recall (*R*) measures the proportion of correctly predicted positive samples relative to all actual positive instances, defined as:

(12) $$Recall=\frac{TP}{FP+FN},$$

where *TP* denotes the number of true positive instances, *FP* represents false positives, and *FN* indicates false negatives.

#### 4.2.2. *mAP*_50_ and *mAP*_50:95_

Mean Average Precision (mAP) evaluates the area under the Precision-Recall curve and is calculated as follows:

(13) $$AP=\int_{0}^{1}P\left(R\right)dR,$$

(14) $$mAP=\frac{\sum_{i=1}^{n}{AP}_{i}}{n},$$

where *mAP*_50_ corresponds to the mean average precision computed at an Intersection over Union (IoU) threshold of 0.5, while *mAP*_50:95_ represents a more stringent evaluation criterion by averaging precision values across IoU thresholds ranging from 0.5 to 0.95.

#### 4.2.3. Parameter Count, Floating Point Operations (FLOPs) and Frames per Second (FPS)

The total number of parameters refers to the sum of all weights and biases within the deep learning model, directly influencing storage requirements and learning capacity. Generally, larger parameter counts correspond to longer training and inference times.

*FLOPs* serve as a metric for computational complexity, with lower values indicating faster execution speeds. The calculation for convolutional operations is expressed as:

(15) $$FLOPs\left(Conv\right)=\left(2\times C_{in}\times K^{2}-1\right)\times W_{out}\times H_{out}\times C_{out},$$

(16) $$FLOPs(Conv)=\left(2\times C_{in}-1\right)\times C_{out},$$

where $C_{in}$ and $C_{out}$ denote input and output channels, respectively, while K, $H_{out}$, and $W_{out}$ represent the kernel size, output feature map height, and output feature map width.

Frames per second (FPS)—the reciprocal of average inference latency—provides a concise indicator of real-world throughput, capturing the rate at which the network processes input frames under a given hardware setup.

### 4.3. Experimental Environment and Initial Parameter Settings

The experiments are conducted on a Windows 10 operating system, with the software executed in PyCharm 2024.1 (Professional Edition). The deep learning framework utilized includes CUDA 12.4, Python 3.11.0, and PyTorch 2.5.1. The hardware configuration comprises an NVIDIA RTX 4090D GPU with 24 GB of VRAM and an AMD Ryzen 9 7950X CPU operating at 4.50 GHz. The hyperparameter settings for training are presented in Table 1.

### 4.4. Performance Evaluation

To comprehensively evaluate the advancements introduced by the EcoDetect-YOLOv2 model, a comparative analysis was conducted against the baseline YOLOv8s model. The experimental results are summarized in Table 2.

The results, validated using the test dataset, demonstrate that EcoDetect-YOLOv2 achieves an improvement of 1.0% in precision, 4.6% in recall, 4.8% in mAP_50_, and 3.1% in mAP_50:95_, while simultaneously reducing parameter count by 19.3% compared to YOLOv8s.

**Figure 9:**
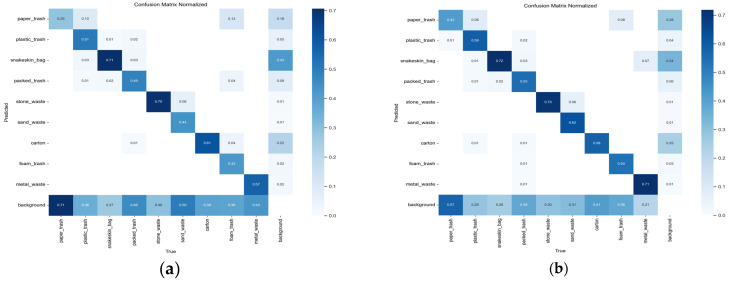
Confusion matrices under the mAP@0.5 metric. (**a**) YOLOv8s; (**b**) EcoDetect-YOLOv2.

Figure 9 illustrates the confusion matrices for the YOLOv8s baseline model and the proposed EcoDetect-YOLOv2 under the mAP_50_ metric, highlighting a substantial enhancement in classification accuracy across nearly all categories. Notably, EcoDetect-YOLOv2 improves the classification accuracy of the frequently misclassified small-target category paper trash by 13%. Additionally, accuracy improvements of 8% and 6% were observed for the small-object classes plastic trash and packed trash, respectively, indicating a superior ability to detect small-scale objects. Furthermore, larger-object categories such as sand waste and metal waste exhibit accuracy gains of 16% and 14%, respectively, demonstrating that the model enhances detection performance across various object scales. Meanwhile, snakeskin bag and stone waste maintain their already high accuracy, with only a slight reduction in carton classification accuracy. Overall, these findings underscore the substantial improvements in predictive accuracy and generalization capabilities achieved by EcoDetect-YOLOv2 compared to the YOLOv8s baseline.

**Figure 10:**
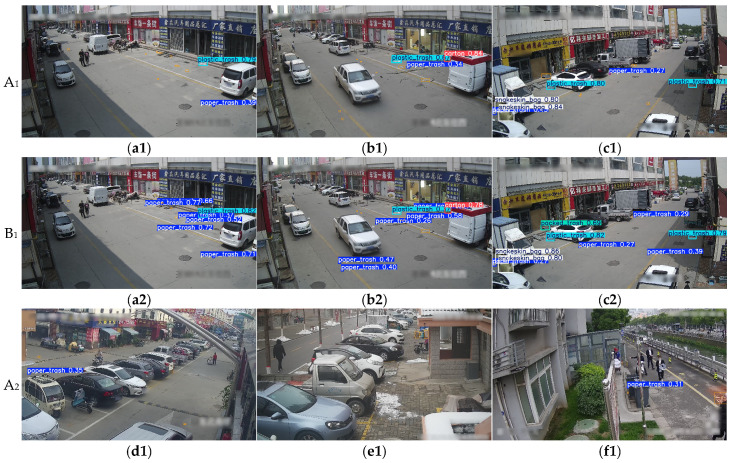

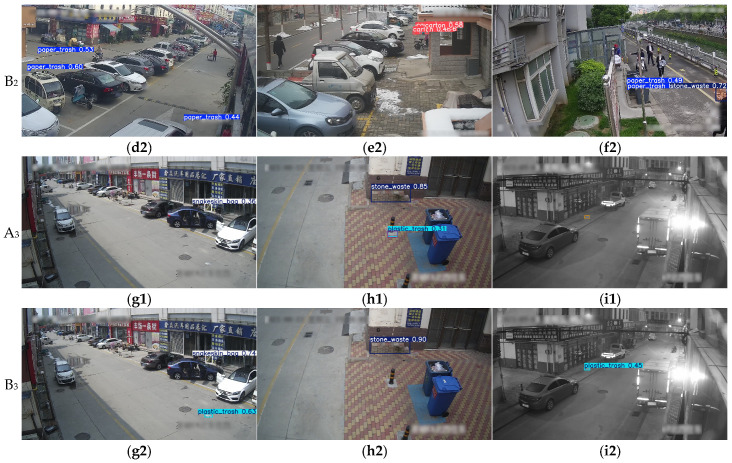
Visual comparison of detection results on the dataset. Row A presents detection outcomes of the baseline YOLOv8 model, while Row B shows results from the proposed EcoDetect-YOLOv2. Bounding box colors indicate different types of detection errors: orange denotes missed detections, purple indicates false positives, and all remaining colors represent correctly identified waste items across different categories. Subpanels (**a**–**f**) compare the two models’ performance on smallobject detection tasks; panels (**c**,**g**,**h**) highlight the baseline model’s elevated false-positive tendency; panel (**i**) illustrates detection efficacy under nighttime conditions.

To further assess EcoDetect-YOLOv2’s performance in garbage exposure detection from surveillance camera viewpoints, qualitative comparisons were conducted using multiple images from the test dataset, as depicted in Figure 10. The experimental results reinforce the limitations of YOLOv8s in complex scenarios.

As illustrated in Figure 10a–f, YOLOv8s exhibits severe misdetection issues in small-target detection scenarios. Specifically, Figure 10a–d shows that due to the minimal size of paper trash objects within the image, YOLOv8s fails to detect almost all instances of this category. In contrast, while EcoDetect-YOLOv2 still exhibits some level of false negatives, it is capable of effectively identifying small paper trash objects. Beyond paper trash, YOLOv8s also exhibits high false negative rates for other distant or small-scale objects. For instance, as shown in Figure 10e, a remotely placed carton is not correctly detected by YOLOv8s, whereas EcoDetect-YOLOv2 successfully identifies it. Similarly, in Figure 10f, stone waste objects with small spatial footprints are missed by YOLOv8s but are successfully detected by EcoDetect-YOLOv2.

Furthermore, YOLOv8s demonstrates significant false-positive detection errors. For example, as shown in Figure 10c, the model incorrectly classifies a faded parking line as paper trash, while in Figure 10g, the foundation of a structure is misclassified as plastic trash. In contrast, EcoDetect-YOLOv2 exhibits superior robustness in such challenging detection environments. Likewise, in Figure 10g, a nearby plastic bag is missed by YOLOv8s yet is recognized with high confidence by EcoDetect-YOLOv2.

Under low-light conditions, the detection performance of YOLOv8s further deteriorates. As shown in Figure 10i, in nighttime scenarios, YOLOv8s fails to detect clearly visible plastic trash objects, whereas EcoDetect-YOLOv2, despite reduced confidence scores, successfully identifies these targets. These experimental findings suggest that EcoDetect-YOLOv2 is well-adapted to variations in target size and feature characteristics, significantly enhancing both detection accuracy and robustness.

### 4.5. Analysis of Attention Mechanisms

To systematically evaluate the advantages of the Exponential Moving Average (EMA) attention mechanism, multiple mainstream attention mechanisms were integrated into the YOLOv8s model for comparative analysis, including Coordinate Attention (CA) [31], Squeeze-and-Excitation (SE) [32], NonlocalBlockND [33], Convolutional Block Attention Module (CBAM) [33], and Spatial-Channel Synergic Attention (SCSA) [34]. These evaluations aim to determine the effectiveness of each attention mechanism within the context of this task.

CA enhances feature spatial encoding by incorporating position sensitivity and directional awareness, thereby improving object localization accuracy. SE dynamically adjusts channel weights based on their importance to mitigate information loss caused by imbalanced channel weight allocation. NonlocalBlockND captures long-range dependencies by computing correlations between arbitrary positions in the feature map, enhancing global contextual understanding. However, its high computational complexity increases processing overhead when handling high-resolution feature maps. CBAM integrates both channel and spatial attention mechanisms, optimizing feature channel information allocation through global average pooling and max pooling, while its spatial attention module highlights critical regions by compressing channel dimensions. Despite its advantages in improving detection performance, CBAM’s computational efficiency is slightly lower than that of lightweight attention mechanisms such as EMA. SCSA overcomes traditional attention mechanisms’ limitations in local and global feature modeling by leveraging spatial-channel synergistic modeling and multi-semantic information fusion.

As shown in Table 3, integrating EMA into YOLOv8s yields the highest overall detection performance. Compared to the original YOLOv8s, EMA achieves precision, recall, mAP_50_, and mAP_50:95_ values of 68.9%, 53.5%, 57.3%, and 33.3%, respectively, with both mAP metrics attaining optimal performance. Although EMA does not achieve the highest individual precision or recall values, it provides the best balance between these two metrics, enhancing model robustness in detection tasks. Moreover, the introduction of EMA does not significantly increase computational complexity or parameter size, further validating its effectiveness in improving object detection performance while maintaining a lightweight model structure.

### 4.6. Results of Ablation Experiments

To evaluate the effectiveness of the proposed modifications in EcoDetect-YOLOv2, a systematic ablation study was conducted (Table 4). Model 0 corresponds to the baseline YOLOv8s, while Models 1 to 15 represent different combinations of modifications, enabling an assessment of each component’s impact on detection performance. In Table 4, a tick (“T”) signifies the inclusion of the respective algorithmic enhancement and a dash (“-”) indicates its omission.

The results in Table 4 highlight several key findings:

(1) Impact of the P2 Small-Object Detection Layer: Model 1 demonstrates that integrating the P2 small-object detection layer enhances detection performance in surveillance camera scenarios, where small-object detection is crucial. The precision (P), recall (R), mAP_50_, and mAP_50:95_ increased by 2.2%, 1.9%, and 0.4%, respectively. Despite a slight decrease in precision, this aligns with the precision-recall trade-off, where increased recall and detection coverage may result in some false positives. However, from a holistic perspective, this trade-off is acceptable and contributes to overall model robustness.
(2) Effectiveness of the EMA Module: Model 2 confirms that the inclusion of the EMA module significantly enhances detection capability. The EMA module leverages cross-spatial learning to reshape part of the channel dimensions into batch dimensions, processing them in groups. By integrating 3×3 convolutions at multiple scales, it enables joint modeling of channel and spatial information. The parallel sub-network structure applies multi-scale convolution to grouped sub-features, suppressing background noise and enhancing target response through a dynamic modulation mechanism while avoiding dimensional reduction issues common in traditional attention mechanisms. Furthermore, the cross-spatial feature aggregation strategy of EMA, coupled with lightweight decomposition and depthwise separable convolution, reduces computational complexity while strengthening the representation of small objects. Experimental results indicate that the EMA module improves P, R, mAP_50_, and mAP_50:95_ by 0.7%, 1.4%, 2.2%, and 1.5%, respectively, demonstrating its effectiveness in complex scenarios.
(3) Contribution of DySample: Model 3 reveals that incorporating DySample results in significant improvements in R, mAP_50_, and mAP_50:95_ (increases of 1.0%, 0.8%, and 0.7%, respectively). DySample enhances multi-scale feature representation through dynamic upsampling and lightweight decomposition while introducing minimal additional parameters. Although P slightly decreases from 68.2% to 67.8%, this may be attributed to DySample’s increased feature sensitivity, which lowers the response threshold for edge-blurred regions and background noise. Nevertheless, overall performance gains indicate that DySample effectively balances efficiency and robustness by leveraging cross-scale feature aggregation and dynamic sampling point segmentation.
(4) Impact of the ResGhostCSP Module: Results from Model 4 demonstrate that the proposed ResGhostCSP, based on GhostConv, significantly reduces computational complexity while maintaining detection performance. This enables simultaneous optimization of model lightweighting and detection accuracy.
(5) Combinatorial Optimization of Multiple Components: Models 5 through 14 represent experiments combining different enhancement modules. While the inclusion of multiple modifications slightly increases computational complexity and parameter count, the overall detection accuracy improves. Notably, the introduction of ResGhostCSP effectively reduces computational costs while preserving detection performance, validating the efficacy of multi-component collaborative optimization.
(6) Overall Performance of EcoDetect-YOLOv2: Model 15, the proposed final model in this study, exhibits superior performance in the given task. Compared with the baseline YOLOv8s, it achieves improvements of 0.5% in P, 10.9% in R, 9.0% in mAP_50_, and 6.1% in mAP_50:95_. Additionally, the parameter count is reduced by 19.3%, and computational complexity experiences only a slight increase. These results confirm that EcoDetect-YOLOv2 effectively enhances waste detection under surveillance camera perspectives while maintaining a lightweight structure. The findings offer an efficient and reliable solution for intelligent environmental monitoring and digital governance.

### 4.7. Comparison of Different Methods

To evaluate the performance of EcoDetect-YOLOv2, a comparative analysis was conducted against various state-of-the-art object detection models of comparable scale, including YOLOv3-tiny, YOLOv5s, YOLOv8s, and YOLO11s, as well as the medium (m) and large (l) variants of the YOLOv5, YOLOv8, and YOLO11 series. Additionally, several advanced detection models, such as Waste-YOLO, YOLOv5s-OCDS, EcoDetect-YOLO, and GCC-YOLO, were included in the comparison. All models were trained under identical experimental conditions and hyperparameter settings, with the results presented in Table 5.

As presented in Table 5, EcoDetect-YOLOv2 outperforms existing object detection models in terms of recall (R), mean Average Precision at IoU 0.5 (mAP_50_), and mean Average Precision at IoU 0.5:0.95 (mAP_50:95_), despite operating with reduced computational complexity and parameter count. While the precision (P) is not the highest among the models, the overall detection performance remains superior.

Compared to models of similar scale, including YOLOv3-tiny, YOLOv5s, YOLOv8s, and YOLO11s, EcoDetect-YOLOv2 demonstrates enhanced detection capability, likely due to the improved robustness against background interference and superior small-object detection. Conversely, YOLOv8n, despite its minimal computational complexity, exhibits limited detection performance, making it unsuitable for effective waste detection.

As the model size and complexity increase, detection performance improves across the larger variants of the YOLOv5, YOLOv8, and YOLO11 series (including the m and l variants), as well as the SSD and RTDETR series (comprising the l and x variants). However, the performance enhancements become marginal in relation to the additional computational cost incurred. Notably, YOLO11l demonstrates a lower precision compared to YOLO11m, indicating that excessive model complexity may contribute to overfitting.

Compared with state-of-the-art models such as GCC-YOLO, Waste-YOLO, and YOLOv5s-OCDS, EcoDetect-YOLOv2 achieves the highest recall and mAP scores, demonstrating enhanced robustness in challenging scenarios. In summary, EcoDetect-YOLOv2 achieves optimal detection performance while maintaining relatively low computational complexity, highlighting its efficiency and robustness for real-world waste detection applications.

## 5. Conclusions

This study addresses the challenge of accurately identifying and localizing multi-scale waste objects within complex backgrounds from surveillance camera perspectives by introducing a lightweight and efficient detection model, EcoDetect-YOLOv2. Built upon YOLOv8s, the model integrates a P2 small-object detection layer and an efficient multi-scale attention (EMA) mechanism, significantly enhancing small-object detection capabilities while improving robustness in complex environments and noise conditions. The adoption of the Dysample upsampling technique optimizes feature fusion and improves discrimination of overlapping objects. Furthermore, the incorporation of GhostConv and the one-shot aggregation-based ResGhostCSP module effectively reduces computational complexity and inference time while maintaining detection accuracy.

Experimental results indicate that, despite a 19.3% reduction in parameter count, EcoDetect-YOLOv2 achieves a 1.0%, 4.6%, 4.8%, and 3.1% improvement in precision, recall, mAP_50_, and mAP_50:95_, respectively, compared to the baseline YOLOv8s model. These findings validate the model’s capability for real-time multi-object waste detection in surveillance applications. Overall, EcoDetect-YOLOv2 provides a robust and efficient solution for automated urban waste management and the advancement of digital economy applications. Future research will focus on leveraging collaborative learning [38] and transfer learning strategies to enhance model’s adaptability to larger datasets and more complex environments, as well as optimizing real-time system integration, further driving the progress of intelligent waste management systems.

## Figures and Tables

**Table 1:** Hyperparameter settings for training.

Parameter Name	Parameter Value
Epochs	300
Batch size	16
Image size	640
Initial learning rate	0.01
Momentum	0.937
Weight decay	0.0005
Optimizer	SGD

**Table 2:** Performance Comparison Between YOLOv8s and EcoDetect-YOLOv2 Models.

Models	P	R	mAP_50_	mAP_50:95_	FLOPs (G)	Parameters (MB)
YOLOv8s	68.2	52.1	55.1	31.8	23.6	9.39
Our EcoDetect-YOLOv2	69.2	56.7	59.9	34.9	26.2	7.58

**Table 3:** Comparison of Attention Mechanism Results.

Attention	P	R	mAP_50_	mAP_50:95_	FLOPs(G)	Parameters (MB)
None	68.2	52.1	55.1	31.8	23.6	9.39
SE	68.9	51.1	55.6	31.3	23.6	9.39
CA	68.2	53.0	56.7	32.6	23.6	9.39
NonlocalBlockND	68.2	53.6	56.5	32.4	24.2	9.54
CBAM	69.6	50.8	56.4	32.1	23.8	9.63
SCSA	69.1	51.7	56.9	32.5	23.6	9.39
EMA	68.9	53.5	57.3	33.3	23.7	9.39

**Table 4:** Results of ablation experiments.

Num	P2	EMA	Dysample	RGC	P	R	mAP_50_	mAP_50:95_	FLOPs (G)	Parameters (MB)
0	-	-	-	-	68.2	52.1	55.1	31.8	23.6	9.39
1	T	-	-	-	67.5	54.3	57.0	32.2	31.7	9.56
2	-	T	-	-	68.9	53.5	57.3	33.3	23.7	9.39
3	-	-	T	-	67.8	53.1	55.9	32.5	23.6	9.41
4	-	-	-	T	68.6	51.8	55.3	31.7	19.8	7.49
5	T	T	-	-	70.2	55.6	58.9	34.3	31.8	9.57
6	T	-	T	-	67.6	55.0	57.6	32.9	31.8	9.59
7	T	-	-	T	67.7	54.1	57.1	32.4	26.1	7.58
8	-	T	T	-	68.4	53.7	57.8	33.5	23.7	9.41
9	-	T	-	T	69.1	53.0	57.4	33.2	19.8	7.50
10	-	-	T	T	68.0	52.7	56.2	32.5	23.6	9.41
11	T	T	T	-	69.6	56.6	59.6	34.9	31.8	9.59
12	T	T	-	T	70.4	55.4	59.0	34.2	26.2	7.58
13	T	-	T	T	68.1	54.7	57.8	33.0	26.2	7.58
14	-	T	T	T	68.7	53.6	57.9	33.3	19.8	7.50
15	T	T	T	T	69.2	56.7	59.9	34.8	26.2	7.58

**Table 5:** Performance comparison of different models.

Model	P	R	mAP_50_	mAP_50:95_	FLOPs (G)	Parameters (MB)	FPS
SSD	65.1	47.7	52.5	29.8	268.5	23.8	112.44
DETR	-	-	38.5	11.9	86.0	41.0	299.61
RT-DETR-l	60.7	44.9	49.0	28.0	103.9	30.54	161.12
RT-DETR-x	65.1	42.4	46.0	25.0	223.2	62.49	134.31
YOLOv3	68.9	52.1	57.3	32.1	154.7	58.7	196.77
YOLOv3-tiny	53.3	39.9	45.8	27.7	12.9	8.3	967.48
YOLOv5s	68.6	50.1	52.4	30.1	15.8	6.7	638.73
YOLOv5m	69.1	52.6	55.2	32.6	48.0	19.9	411.48
YOLOv5l	69.4	53.5	56.5	33.0	107.8	44.0	308.59
YOLOv8n	55.4	44.9	48.6	28.9	6.9	2.56	758.13
YOLOv8s	68.2	52.1	55.1	31.8	23.6	9.39	601.56
YOLOv8m	69.1	52.6	56.3	32.7	67.8	22.15	354.87
YOLOv8l	67.4	55.5	57.0	33.1	145.8	37.64	294.11
YOLO11s	68.2	52.1	55.1	31.8	21.5	8.99	627.19
YOLO11m	69.9	53.4	56.9	33.2	68.2	19.13	407.62
YOLO11l	69.0	53.5	57.0	33.5	87.3	24.14	306.85
GCC-YOLO [35]	70.1	54.7	57.3	32.3	19.8	7.2	623.47
Waste-YOLO [36]	67.1	50.5	53.7	30.3	16.7	6.71	606.47
YOLOv5s-OCDS [37]	67.5	51.1	56.2	30.9	54.2	13.81	467.33
EcoDetect-YOLO [18]	73.5	52.9	58.3	31.2	20.0	7.0	589.12
Our EcoDetect-YOLOv2	69.2	56.7	59.9	34.8	26.2	7.58	553.97

## Data Availability

The datasets used and analyzed during the current study are available from the corresponding author upon reasonable request.
